# Diurnal Variation of Hepatic Antioxidant Gene Expression in Mice

**DOI:** 10.1371/journal.pone.0044237

**Published:** 2012-08-29

**Authors:** Yi-Qiao Xu, Dan Zhang, Tao Jin, Ding-Jun Cai, Qin Wu, Yuanfu Lu, Jie Liu, Curtis D. Klaassen

**Affiliations:** 1 Zunyi Medical College, Zunyi, China; 2 Chengdu University of Traditional Chinese Medicine, Chengdu, China; 3 University of Kansas Medical Center, Kansas City, Kansas, United States of America; National Institutes of Health, United States of America

## Abstract

**Background:**

This study was aimed to examine circadian variations of hepatic antioxidant components, including the Nrf2- pathway, the glutathione (GSH) system, antioxidant enzymes and metallothionein in mouse liver.

**Methods and Results:**

Adult mice were housed in light- and temperature-controlled facilities for 2 weeks, and livers were collected every 4 h during the 24 h period. Total RNA was isolated, purified, and subjected to real-time RT-PCR analysis. Hepatic mRNA levels of Nrf2, Keap1, Nqo1 and Gclc were higher in the light-phase than the dark-phase, and were female-predominant. Hepatic GSH presented marked circadian fluctuations, along with glutathione *S*-transferases (GST-α1, GST-µ, GST-π) and glutathione peroxidase (GPx1). The expressions of GPx1, GST-µ and GST-π mRNA were also higher in females. Antioxidant enzymes Cu/Zn superoxide dismutase (Sod1), catalase (CAT), cyclooxygenase-2 (Cox-2) and heme oxygenase-1 (Ho-1) showed circadian rhythms, with higher expressions of Cox-2 and CAT in females. Metallothionein, a small non-enzymatic antioxidant protein, showed dramatic circadian variation in males, but higher expression in females. The circadian variations of the clock gene Brain and Muscle Arnt-like Protein-1(Bmal1), albumin site D-binding protein (Dbp), nuclear receptor Rev-Erbα (Nr1d1), period protein (Per1 and Per2) and cryptochrome 1(Cry1) were in agreement with the literature. Furthermore, acetaminophen hepatotoxicity is more severe when administered in the afternoon when hepatic GSH was lowest.

**Conclusions:**

Circadian variations and gender differences in transcript levels of antioxidant genes exist in mouse liver, which could affect body responses to oxidative stress at different times of the day.

## Introduction

Mammals have developed an endogenous circadian clock located in the suprachiasmatic nucleus of the anterior hypothalamus that responds to the solar time [1]. Human homeostatic systems have adapted to daily changes in a way that the body anticipates the sleep and activity periods. Similar clocks have been found in peripheral tissues, such as the liver [2]–[3]. The liver is the major organ of drug metabolism and detoxification, the circadian variation of the hepatic defense system can alter the balance between therapeutic response and toxicity [3]–[5]. The complexity of tissue- and day-time specific regulation of thousands of clock controlled genes also involves many transcriptional regulators [6].

Circadian rhythms control myriads of physiological processes, including the production of reactive oxygen species (ROS) and its elimination, redo-sensitive gene activation and depression [7]. Endogenous circadian and exogenously driven daily rhythms include antioxidant enzymes and low molecular weight antioxidants such as glutathione [8]. Diurnal fluctuations of glutathione (GSH) have been shown in animals [9] and in humans [10]. Circadian variation of hepatic GSH-*S* transferase (GST) activity is also evident in rodents [11]. Catalase activity in mouse organs also showed rhythmic patterns [12]. Day/night rhythms in liver lipid peroxidation levels exist, and resveratrol could function as a pro-oxidant during daytime, but antioxidant at night [13]. Cu/Zn superoxide dismutase is differentially regulated in Period gene mutant mice [14]. The nuclear factor erythroid-2-related factor-2 (Nrf2) and cyclooxygenase 2(Cox-2) confers protection against oxidative stress [15], and may follow the rhythm pattern of melatonin [16].

Circadian rhythms of the defense machinery could affect the sensitivity to environmental stimuli. For example, the hepatotoxicity of carbon tetrachloride was observed to be greater when administered in the afternoon [17]. The clock gene Period2 (per2)-knockout mice are sensitive to carbon tetrachloride-induced hepatotoxicity [18], but are less sensitive to acetaminophen hepatotoxicity when injected at night-time (20∶00) with a decreased expression of Cyp1a2, a P450 enzyme gene implicated in acetaminophen bioactivation [19]. Thus, chronotoxicity of acetaminophen has been shown to correlate with circadian rhythms in metabolism genes to activate or to detoxify acetaminophen metabolites [20]. The chronopharmacological lethal effect of lipopolysaccharide (LPS) may also relate to the time-dependent increase of serum TNFα level and simultaneously high level of Per2 gene expression in the heart and liver between ZT12-18 of the day [21].

The previous study provoked us to make a systemic investigation of diurnal variations of these common antioxidant components in mouse liver. We initially verified the circadian rhythms of 6 clock genes, followed by examination of circadian variations of the Nrf2 antioxidant pathways, hepatic glutathione (GSH) and GSH-related enzymes, the antioxidant enzymes, and a small non-enzymatic protein metallothionein, and described circadian rhythms of these antioxidant gene expressions, which could impact the body defense against various toxic stimuli.

## Materials and Methods

### Ethics statement

All animal procedures follow the NIH Guide of Humane Use and Care Animals. Mice were housed maintained in the SPF-grade animal facilities at Zunyi Medical College, China. All procedures were carried out in compliance with standards for use of laboratory animals and have been approved by the Institutional Animal Care and Use Committee.

### Animals

Male and female outbred Kunming (KM) mice (6 weeks of age) were obtained from the Animal Breeding Center (Chongqing, China), and housed for 2 weeks in a temperature-humidity controlled facility with a standard 12 h (8∶00–20∶00) light schedule. Mice had free access to rodent chow and drinking water. Mice (n = 4/time point) were anesthetized and tissues were harvested at 2∶00, 6∶00, 10∶00 am and 14∶00, 18∶00 and 22∶00, and frozen in liquid nitrogen and stored at −80°C prior to analysis.

### RNA Isolation and Real-time RT-PCR analysis

Total RNA was extracted from frozen liver sample (50–100 mg) using 1 ml TRIzol (Invitrogen, Carlsbad, CA) and subsequently purified with RNeasy columns (Qiagen, Valencia, CA). The quality and integrity of purified RNA was determined by spectrophotometry and agarose gel electrophoresis respectively. Reverse transcript reaction according to the manufacturer's instruction of High Capacity Reverse Transcriptase Kit (Applied Biosystems, Foster City, CA, USA). The primer pairs were designed with the Primer3 software and are listed in Table S1. The Power SYBR Green Master Mix (Applied Biosystems, Foster City, CA, USA) was used for real-time RT-PCR analysis. The β-actin and G3PDH housekeeping genes were used as percentage controls for normalization.

### Hepatic GSH determination

Hepatic GSH content was determined with a GSH assay kit (Nanjing, China) following the manufacturer's protocol. Briefly, approximately 100 mg of liver tissue was homogenized in 0.9 ml physiological saline, followed by centrifugation (2500 rpm for 10 min). Aliquots of the supernatant (0.1 ml) were mixed with an equal volume of precipitation reagent, and centrifuged (2500 rpm for 10 min). The supernatant (0.1 ml) was mixed with 30% hydrogen peroxide (100 µl) and TMB (3,3′,5,5′-tetramethylbenzidine, 25 µl), and the absorbance was determined at 405 nm in a 96-well plate.

### Hepatic MT protein determination

Hepatic MT protein was determined by the cadmium–hemoglobin assay. Liver tissues were homogenized in physiological saline (1∶10, wt: vol), followed by centrifugation at 10,000 g for 10 min. Aliquots of the supernatant (0.1 ml) was mixed with CdCl_2_ solution (2 µg Cd/ml;100 µl), followed by adding 50 µl of 2% hemoglobin. The mixture was heated in boiling water for 90 seconds and then centrifuged at 12,000 g for 5 min. Another 50 µl of 2% hemoglobin was added, boiled and centrifuged again. The supernatant (100 µl) was taken for determination of Cd by the absorption of atoms spectrophotometer (VARIAN, Mississauga, ON,Canada).

### Acetaminophen hepatotoxicity

Mice were given an i.p. injection of acetaminophen (500 mg/kg in 20 ml/kg saline) at 6∶00 and 18∶00, and blood was collected 12 hrs later for determination of serum activity of alanine aminotransferase (ALT) and aspartate aminotransferase (AST) using a colorimetric kit (Shanghai Diagnostics, China).

### Statistics

Date were described using the mean ± SEM and analyzed by the one-way ANOVA. Circadian variation was analyzed by the intergroup average cosine algorithm method. Sex difference and acetaminophen hepatotoxicity were analyzed by the student's *t*-test. P<0.05 was considered statistically significant.

## Results

### Circadian variations of clock gene mRNA in the liver of mice

**Figure 1:**
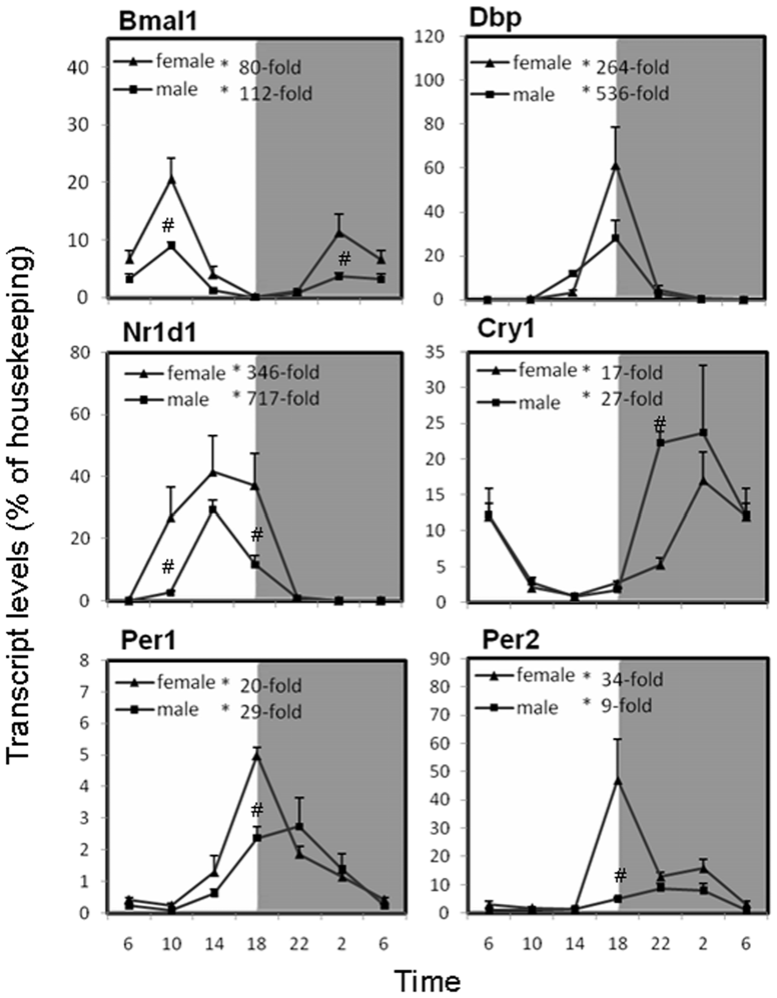
Circadian variations of mRNA levels of the clock gene Brain and Muscle Arnt-like Protein-1(Bmal1), albumin site D-binding protein (Dbp), nuclear receptor Rev-Erbα (Nr1d1), cryptochrome 1 (photolyase-like) (Cry1), period protein 1 (Per1) and period protein 2 (Per2) in adult female and male mouse livers (n = 4 for each time point). *Significant circadian rhythm p<0.05; #Significant sex difference p<0.05.

The mRNAs of major clock genes tended to accumulate more in the light phase. As shown in Fig. 1, Brain and Muscle Arnt-like Protein-1(Bmal1) mRNA displayed a robust circadian rhythm with a peak/trough ratio of 80 for females, 112 for males; with the highest expression occurred at 10∶00 and the lowest at 18∶00. Albumin site D-binding protein (Dbp), period protein 1 (Per1) and period protein 2 (Per2) had the highest mRNA levels at 18∶00, with peak/trough ratios of 264, 20 and 34 for females, 536, 29 and 9 for males, respectively. Nuclear receptor Rev-Erbα (Nr1d1) mRNA had the nadir and peak differences of 346 for females and 717 for males, and the higher expression appeared during the late light phase. Only cryptochrome 1(Cry1) mRNA exhibited marked fluctuation with a 17-fold peak/trough ratio for females and 27-fold peak/trough radio for males during the dark phase. These clock gene expression patterns indicate that the outbred KM mouse livers had typical circadian rhythms and were valid for further investigations. Interestingly, except for Cry1 and Dbp, other four clock genes were highly expressed in females.

### Circadian variations of Nrf2-pathway mRNA in the liver of mice

**Figure 2:**
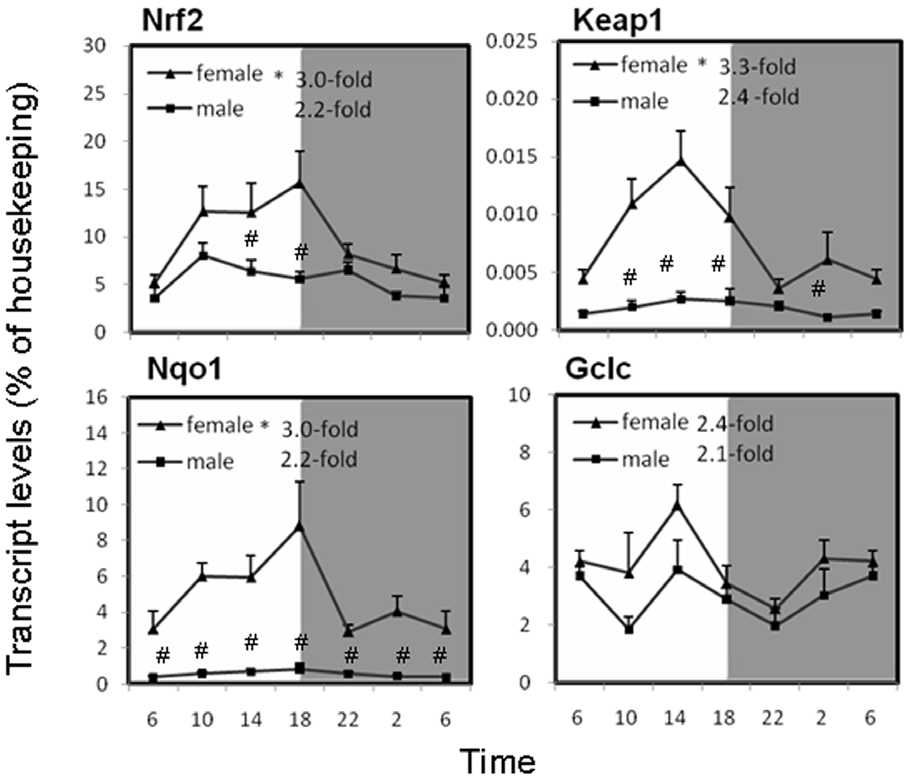
Circadian variations of mRNA levels of the Nrf2 pathway-related genes, including the nuclear factor erythroid-2-related factor-2 (Nrf2), Kelch-like ECH associating protein 1 (Keap1), NADPH quinone oxidase 1 (Nqo1), and glutamate-cysteine ligase catalyze subunit (Gclc) in adult female and male mouse livers (n = 4 for each time point). *Significant circadian rhythm p<0.05; #Significant sex difference p<0.05.

The circadian variations of the Nrf2 pathway genes, namely nuclear factor erythroid2-related factor 2 (Nrf2), Kelch-like ECH-associated protein 1(Keap1), quinone oxidoreductase 1 (Nqo1) and glutamate cysteine ligase catalytic subunit (Gclc), are illustrated in Fig. 2. These four genes relatively displayed higher during the light phase. The mRNA of Nrf2 and Nqo1 reached highest expression at 18∶00, then rapidly declined to the lowest level in the early dawn phase (2∶00–6∶00), and returned to a high level in the light phase. The rhythm ratios were 3-fold for females and 2-fold for males. The daily changes in Keap1 and Gclc mRNA were 3.3-fold and 2.4-fold for females, 2.4-fold and 2.2-fold for males, with the peaks at 14∶00 and the nadirs at 22∶00, and circadian variation of Keap1 was significant. The expression of Nrf2-pathway genes except for Gclc was higher in females than in males, especially for Keap1 and Nqo1.

### Circadian variations of GSH and GSH-related gene expression in the liver of mice

**Figure 3:**
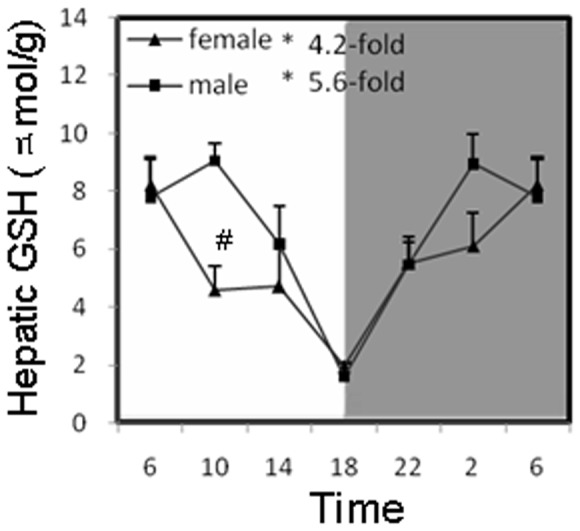
Circadian variations of hepatic glutathione (GSH) in adult female and male mouse livers (n = 4 for each time point). *Significant circadian rhythm p<0.05; #Significant sex difference p<0.05.

**Figure 4:**
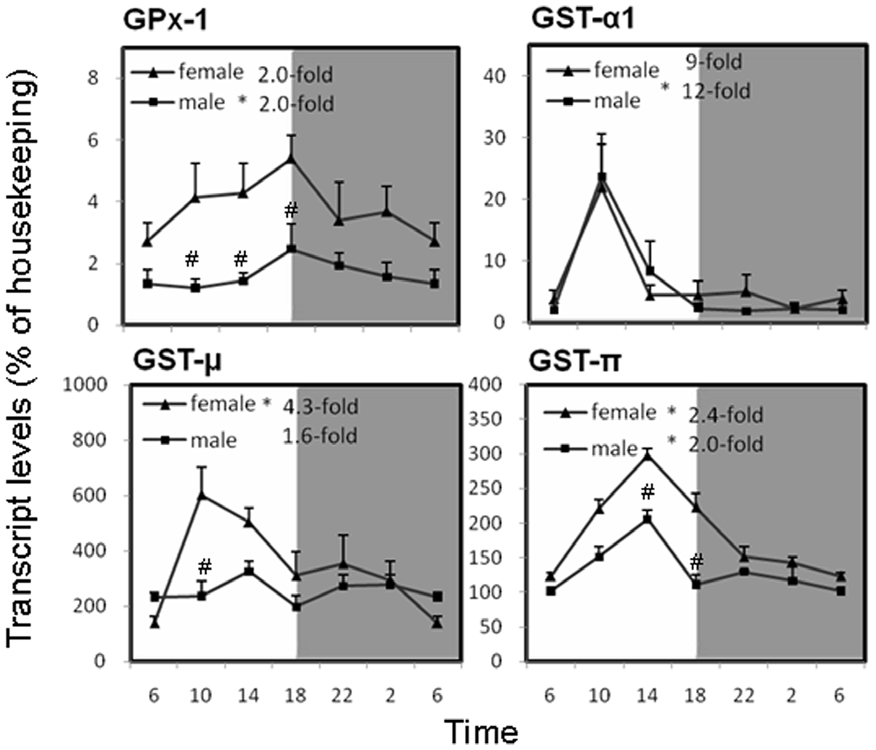
Circadian variations of hepatic GSH-related genes (Gpx1, GST-α1, GST-µ, GST-π) in adult female and male mouse livers (n = 4 for each time point). *Significant circadian rhythm p<0.05; #Significant sex difference p<0.05.

Hepatic GSH content presented a marked circadian fluctuation of a 4.2-fold peak/trough ratio for females and a 5.6-fold peak/trough ratio for males (Fig. 3), and the sex-difference was not appreciable. GSH-related genes, such as Gpx1 and GST-α1 in male, GST-µ in female, GST-π in both genders, also showed an oscillation in mouse liver, with higher expressions in females for Gpx1, GST-µ and GST-π (Fig. 4). Gpx1 is the rate-limiting enzyme in the production of Glutathione oxidized (GSSG). Messenger RNA of Gpx1 oscillated with a 2-fold magnitude and the culmination located at 18∶00. The GST enzyme family carries out a diversity of metabolic functions, including detoxification. In general, the GSTs mRNAs tended to be higher in the light phase, and the variations were greater than 2-fold, and GST-π showed 2.4-fold in females and 2-fold in males. In both females and males, GST-α1 and GST-π peaked at 10∶00 and 14∶00 respectively, while female GST-µ peaked at 10∶00 and male peaked at 14∶00. Except for GST-α1, all the GSH-related genes were higher in females.

### Circadian variations of antioxidant enzyme gene expression in the liver of mice

**Figure 5:**
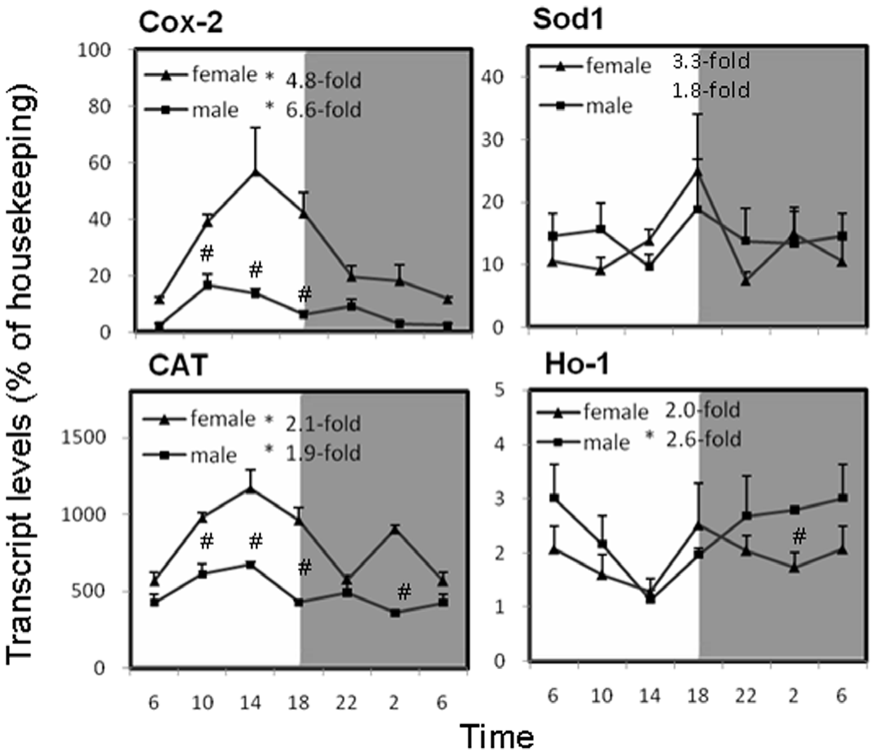
Circadian variations of cycloooxygenae-2 (Cox-2), antioxidant enzymes Cu/Zn superoxide dismutase (Sod1), catalase (CAT), and heme oxygenase-1 (Ho-1) in adult female and male mouse livers (n = 4 for each time point). *Significant circadian rhythm p<0.05; #Significant sex difference p<0.05.

We observed a rhythmic expression of antioxidant enzymes in Fig. 5. Cox-2 displayed the highest fluctuation among the antioxidant genes, with a peak at 14∶00 (the peak/trough ratio was 4.8 for females, 6.6 for males). Catalase (CAT) also showed diurnal variation. Superoxide dismutase 1 (Sod1) and heme oxygenase 1 (Ho-1) transcripts displayed mild fluctuations in mRNA levels over time, with slightly changes at the light phase. Expression of Sod1 and Ho-1 showed the peak/trough values of 3.3 and 2.0 for females and 1.8 and 2.6 for males, respectively. There were no gender differences in expression of Sod1 and Ho-1 mRNA, whereas females expressed significantly more Cox-2 and CAT mRNA than males.

### Circadian variations of Mt mRNA in the liver of mice

**Figure 6:**
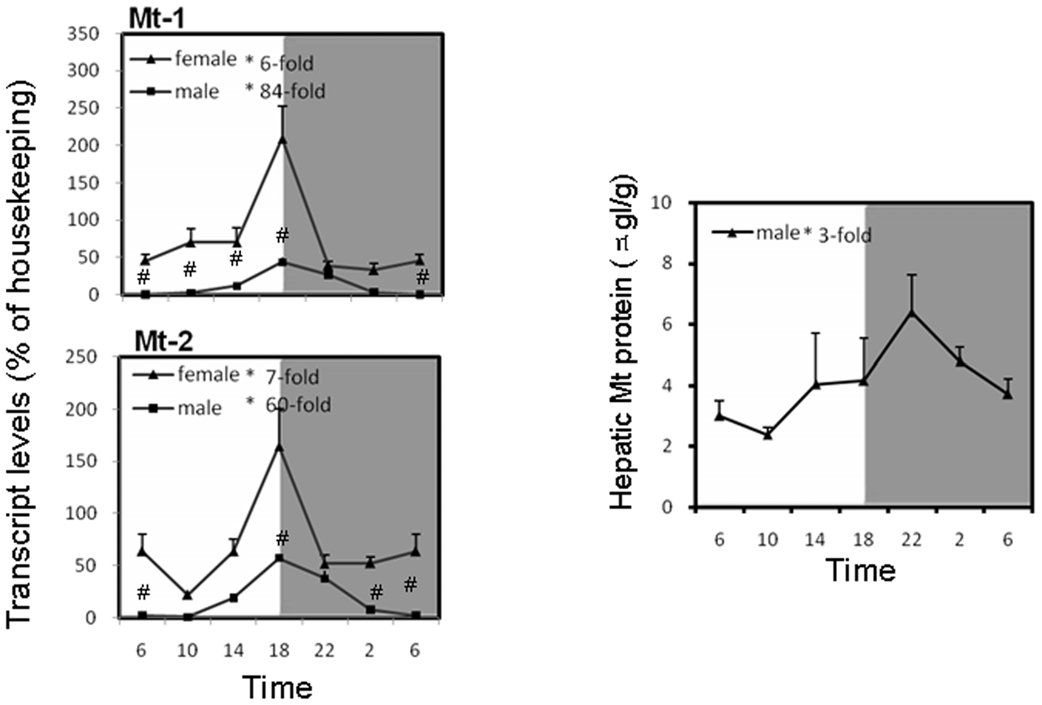
Circadian variations of the small antioxidant protein metallothionein gene (Mt-1 and Mt-2) in adult female and male mouse livers (n = 4 for each time point). *Significant circadian rhythm p<0.05; #Significant sex difference p<0.05.

Mt-1 and Mt-2 are two isoforms highly expressed in mouse liver. Mt-1 exhibited an approximately 6-fold decrease in mRNA between 18∶00 and 22∶00 in females, and 84-fold increase from 6∶00 to 18∶00. Similar to Mt-1, Mt-2 demonstrated nearly 7-fold and 60-fold increases in mRNA levels from 10∶00 to 18∶00 for females and males, respectively (Fig. 6). Females had higher basal levels of Mt transcripts than males, with less circadian variations than males.

Hepatic MT was determined by the Cd/hemoglobin assay, and showed a peak at 22∶00, with the peak/trough ratio of 3 for males. The peak is 4 h late after the highest point of MT mRNA expression.

### Circadian variations of acetaminophen hepatotoxicity in mice

**Figure 7:**
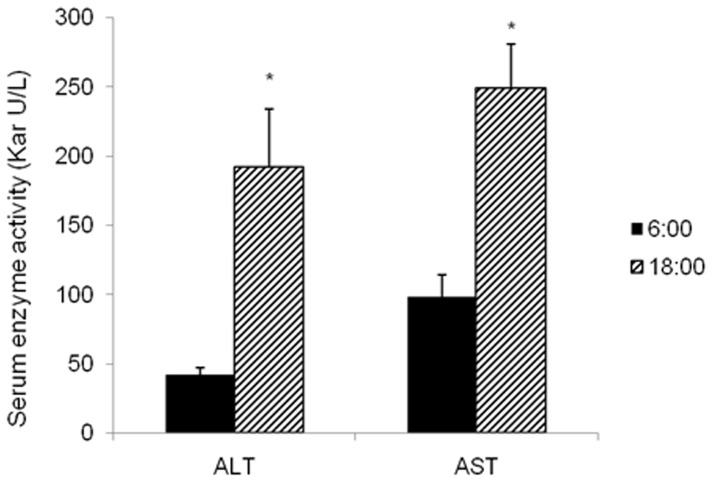
Circadian variation in acetaminophen hepatotoxicity. Mice (n = 10) were administered acetaminophen (500 mg/kg, ip, in 20 ml/saline) at 6∶00 and 18∶00, respectively. Hepatotoxicity was evaluated by serum activities of alanine aminotransferase (ALT) and aspartate aminotransferase (AST) 12 h after acetaminophen administration. Data are mean ± SEM, *Significant difference between 6∶00 and 18∶00, p<0.05.

Fig. 7 shows the circadian variation of acetaminophen hepatotoxicity in mice. Serum activity of alanine aminotransferase (ALT) and aspartate aminotransferase (AST) were higher when acetaminophen was administered at 18∶00, a time point with lowest GSH levels during the day, indicative of more liver injury. In comparison, less hepatotoxicity of acetaminophen is noted when it was administered at 6∶00.

## Discussion

This study clearly demonstrates that the diurnal variations of hepatic antioxidant components in the mouse liver, including the Nrf2 antioxidant pathways, the hepatic GSH systems, the enzymatic antioxidant components and the small antioxidant protein MT. To our knowledge, this is the first systemic investigation on circadian variations and sex-differences of antioxidant components in the mouse liver.

The core circadian clock is composed of interwoven positive and negative feedback loops, in which two transcription factors, CLOCK and BMAL1, form heterodimers and directly activate the transcription of target genes, for instance, Per, Cry, Dbp and Nr1d1. Polyproteins, these gene products, function as negative regulators, inhibiting the transcription activity of CLOCK-BMAL1 heterodimers [1]–[5], [22].The present work demonstrated the rhythms of the 6 major clock genes in the livers of KM mice (Fig. 1), and the patterns of these gene expression are comparable with that reported in the literature [23], [24], which is essential for the examination of diurnal variations of gene expressions encoding hepatic antioxidant systems.

The Nrf2 antioxidant pathway is an emerging master of the defense against oxidative stress [25]. Nrf2 is a transcription factor that positively regulates the basal and inducible expression of a large battery of cytoprotective genes [26]. Under quiescent conditions, Nrf2 interacts with the actin-anchored protein Keap1, largely localized in the cytoplasm, to maintain low basal expression of Nrf2-regulated genes. However, under oxidative and electrophilic stress conditions, Nrf2 is released from Keap1, and translocates to the nucleus to activate the expression of several dozen cytoprotective genes that enhance cell survival [25]–[27]. The major targeted gene products include proteins that catalyze the reduction of quinones and protect hepatocytes against oxidative stress (Nqo1), and the rate-limiting enzyme in the synthesis of GSH (Gclc) [26]–[27]. The expression of the Nrf2-pathway related genes was higher at daytime than at the night-time. The patterns of Nrf2 and Nqo1 are similar to Dbp, as all of them were highest at 18∶00. The patterns of Keap1 and Gclc are similar to Nr1d1, with the peak around 14∶00. Furthermore, females had approximately 10-fold higher transcript levels of Keap1 and Nqo1 than males, and sex dimorphism also exist for Nrf2 and Gclc (Fig. 2). The pineal hormone melatonin acts via G-protein-coupled receptors to synchronize clock-generated circadian rhythms and acts as a paracrine or autocrine agent to control redox-sensitive transcription factors such as NF-κB, AP-1 and Nrf2 [16], and this study further demonstrated the circadian patterns of Nrf2 and Nrf2-related genes in the mouse liver.

The GSH system is the most important defense mechanism against oxidative stress and toxic stimuli. GSH guards the liver against oxidative injury by reducing hydrogen peroxide and scavenging reactive oxygen and nitrogen radicals [28]. Diurnal variations in hepatic GSH content affect the sensitivity to the hepatotoxicity produced by carbon tetrachloride [17] and acetaminophen [29]. In the present study, hepatic GSH content was highest at 6∶00 to 10∶00 and at nadir at 18∶00 in both female and male mice (Fig. 3), consistent with the literature [9] and coincides with the increased toxicity of acetaminophen and carbon tetrachloride when they were administered at the evening time [17], [29]. Glutathione *S*-transferase activities also showed circadian variations, higher in the light phase and lower in the dark phase [11]. In the present study, the transcript levels of GSTs (e.g., GST-α1, GST-µ, GST-π, etc.) were maximally expressed during the light phase and GPx1, GST-µ, GST-π were female predominant (Fig. 4). As a consequence, the rhythmic expression of hepatic GSH and GSH-related genes is important for organisms to fight against various toxic stimuli, particular during the light phase.

The There are two types of antioxidant defense system of the organism, including enzyme reaction system and non-enzymatic system. Antioxidant enzymes, such as Sod, CAT, Cox-2, and Ho-1, produce a slow and long-acting antioxidant and detoxification effects. The major liver antioxidant enzymes Cox-2, a rate-limiting enzyme for the synthesis of prostaglandins, plays important roles in anti-inflammation and oxidative stress [15]. Sod1 converts superoxide anion to hydrogen peroxide, which is further hydrolyzed by CAT. Ho-1 is a rate-limiting enzyme to degrade heme, and is a potent anti-oxidant [30]. These important antioxidant defense mechanisms can be affected by circadian rhythms [8]. Circadian rhythms can not only regulate the body redox status, but also the transcription of redox-sensitive genes [7]. Except for Sod1 (due to big individual variation), the circadian rhythms of other 3 genes were evident, with Cox-2 more obvious (Fig. 5). The expressions of Cox-2 and CAT were also female-predominate. CAT rhythm in mouse brain, kidney and liver has also been observed across a 24-h period [12]. Thus, to profile circadian rhythms of major antioxidant enzyme genes would add to our understanding of body defense against environment stress.

Metallothionein (Mt) is a small cysteine-rich protein that plays important roles in the body defense against environmental stress, not only from metals, but also from oxidative stress produced by chemicals [31]. In mammalians, Mt-1 and Mt-2 are major isoforms ubiquitously distributed in all tissues and play major roles against oxidative stress [31]–[32]. In the present study, circadian variations of Mt-1 and Mt-2 are evident, with the peak at 18∶00, a time point when cellular GSH levels are the lowest, perhaps functioning as a compensatory mechanism to maintain antioxidant capacity of the organism. It should be noted that the differences between the peak and nadir of Mt-1 and Mt-2 expression can be as high as 60–84 fold, strongly suggesting that the expression of this protein is under control by biological clock. The circadian rhythm of Mt is not only evident in the liver, but also in other tissues such as the kidney and blood (data not shown), as well as in other organisms such as *Neurospora crassa* [33]. Circadian variations of metallothionein have been shown to be associated with diurnal variations of sensitivity to cadmium toxicity [34], [35]. Biological significance of circadian variations of Mt warrants further investigation.

In general, females had higher transcript levels of the Nrf2-related genes, the GSH-related enzymes, Cox2, and Mt in the liver, suggesting that females have a higher antioxidant capacity than males. Indeed, a recent report shows that females live longer than males, possibly due to a higher defense mechanism against ROS [36]. Physiological concentrations of estrogens activate estrogen receptors as well as the MAPK and NFκB pathway, which can in turn activate the expression of Sod, glutathione peroxidase and other antioxidant components [36]. The sex dimorphism of hepatic antioxidant components are also likely under circadian clock control, as Cry-/- mice are unable to synthesize CRY1 and CRY2 protein, and cannot sustain sex-differences in some drug metabolic genes [37].

Chronotoxicity is an emerging field in toxicology. For example, chloroform hepatotoxicity is more severe when dosed at 18∶00, a time GSH levels are lowest, as compared the dose time at 10∶00 am [38]. Similarly, carbon tetrachloride toxicity shows the same pattern [17]. In addition to GSH contents, circadian rhythms of CYP2E1 and the production of acetone are also involved in diurnal variations in carbon tetrachloride hepatotoxicity [39], [40], and knockout of the clock gene Per2 make animals more susceptible to carbon tetrachloride [18]. Per2 may function in rhythmicity of acetaminophen hepatotoxicity via altering Cyp1a2 expression in mice [19]. LPS produced time-dependent inhibition of Per1 and Per2 gene expression in the liver, which is implicated in LPS-induced endotoxin shock [21]. In hepatocyte primary cultures, Clk/Clk cells are resistance to diethylnitrosamine (DEN)-induced apoptosis, probably through the lower expression levels of metabolic enzymes for bio-activating DEN [41]. The present study provides the data indicating that acetaminophen hepatotoxicity was much higher when it was administered at 18∶00. Thus, circadian rhythms of hepatic antioxidant components could be a determinant factor affecting body's response to toxic stimuli at different times during the 24 h period.

Emerging evidence indicates the importance of circadian variation of antioxidant components in humans, especially under various pathological conditions including cancer [2]–[5], [7]–[10], [15]. Circadian variation not only affects drug metabolism and therapeutic effects, but also influences the responses to chemotherapy during the different times of the day. The present study is among the first to provide diurnal variations of the Nrf2-related genes and metallothionein, which could help setting appropriate strategy to treat human diseases to reduce side-effects and to avoid drug-resistance resulting from the potential circadian variation.

In summary, the current study clearly characterized the diurnal variations of hepatic antioxidant compartment, including the Nrf2/ARE antioxidant pathways, the GSH systems, the antioxidant enzymes, and the antioxidant protein metallothionein. The circadian variations of these defense mechanisms could be important affecting master responses to oxidative stress.

## Supporting Information

Table S1 **Primer sequences for real-time RT-PCR analysis.**
(DOCX)
