# Supplementary material for: Diurnal Variation of Hepatic Antioxidant Gene Expression in Mice
Source: PLoS One. 2012 Aug 29;7(8):e44237. doi: 10.1371/journal.pone.0044237 (PMC3430632; doi:10.1371/journal.pone.0044237)
Supplement: Table S1 — Primer sequences for real-time RT-PCR analysis. (DOCX) [file pone.0044237.s001.docx]

Table S1. Primer sequences for real-time RT-PCR analysis

| Gene | GenBank | Forward | Reverse |
| --- | --- | --- | --- |
| β-actin | V01217 | TGACCGAGCGTGGCTACAG | GGGCAACATAGCACAGCTTCT |
| Bmal1 | [NM_007489](http://www.ncbi.nlm.nih.gov/sites/entrez?cmd=search&db=nucleotide&dopt=GenBank&term=NM_007489.3) | ACGACATAGGACACCTCGCAGA | CGGGTTCATGAAACTGAACCATC |
| Cox2 | NM_011198 | AGATGCTATCTTTGGGGAGAC | ACATTATTGCAGATGAGAGACTG |
| Cry1 | [NM_007771](http://www.ncbi.nlm.nih.gov/sites/entrez?cmd=search&db=nucleotide&dopt=GenBank&term=NM_007771.3) | GGATCCACCATTTAGCCAGACAC | CATTTATGCTCCAATCTGCATCAAG |
| Dbp | NM_016974 | CTGGCCCGAGTCTTTTTGC | CCAGGTCCACGTATTCCACG |
| G3PDH | M32599 | AACTTTGGCATTGTGGAAGG | GGATGCAGGGATGATGTTCT |
| Gclc | BC019374 | TGGCCACTATCTGCCCAATT | GTCTGACACGTAGCCTCGGTAA |
| Gpx1 | U13705 | GAAGAACTTGGGCCATTTGG | TCTCGCCTGGCTCCTGTTT |
| Gst-α1 | NM_008181 | CGCCACCAAATATGACCTCT | TTGCCCAATCATTTCAGTCA |
| Gst-μ | NM_010358 | CTCCCGACTTTGACAGAAGC | TTGCTCTGGGTGATCTTGTG |
| GST-π | D30687 | TGGGCATCTGAAGCCTTTTG | GATCTGGTCACCCACGATGAA |
| Ho-1 | M33203 | CCTCACTGGCAGGAAATCATC | CCTCGTGGAGACGCTTTACATA |
| Keap1 | NM_016679 | AAGGAACATGATATGCCCTGACA | ACACAGGCCGGCTCCAT |
| Mt-1 | NM_013602 | CTCCGTAGCTCCAGCTTCAC | AGGAGCAGCAGCTCTTCTTG |
| Mt-2 | NM_008630 | CCGATCTCTCGTCGATCTTC | AGGAGCAGCAGCTTTTCTTG |
| Nqo1 | BC004579 | TATCCTTCCGAGTCATCTCTAGCA | TCTGCAGCTTCCAGCTTCTTG |
| Nr1d1 | [NM_145434](http://www.ncbi.nlm.nih.gov/sites/entrez?cmd=search&db=nucleotide&dopt=GenBank&term=NM_145434.3) | GTGAAGACATGACGACCCTGGA | TGCCATTGGAGCTGTCACTGTAG |
| Nrf2 | BC026943 | CGAGATATACGCAGGAGAGGTAAGA | GCTCGACAATGTTCTCCAGCTT |
| Sod1 | NM_011434 | TGGTGGTCCATGAGAAACAA | GTTTACTGCGCAATCCCAAT |
| Per1 | NM_002616 | AGGTACCTGGAGAGCTGCAA | TTCTTGGTCCCCACAGAGAC |
| Per2 | [NM_011066](http://www.ncbi.nlm.nih.gov/sites/entrez?cmd=search&db=nucleotide&dopt=GenBank&term=NM_011066.3) | CCTACAGCATGGAGCAGGTTGA | TTCCCAGAAACCAGGGACACA |
